# Current perspectives and trends of the research on hypertensive nephropathy: a bibliometric analysis from 2000 to 2023

**DOI:** 10.1080/0886022X.2024.2310122

**Published:** 2024-02-12

**Authors:** Lan Wang, Jingyu Wang, Yuemiao Zhang, Hong Zhang

**Affiliations:** a Renal Division, Department of Medicine, Peking University First Hospital, Beijing, China; b Institute of Nephrology, Peking University, Beijing, China; c Key Laboratory of Renal Disease, Ministry of Health of China, Beijing, China; d Key Laboratory of Chronic Kidney Disease Prevention and Treatment, Peking University, Ministry of Education, Beijing, China

**Keywords:** Hypertensive nephropathy, chronic kidney disease, renal fibrosis, bibliometric analysis, research progress

## Abstract

Hypertensive nephropathy continues to be a major cause of end-stage renal disease and poses a significant global health burden. Despite the staggering development of research in hypertensive nephropathy, scientists and clinicians can only seek out useful information through articles and reviews, it remains a hurdle for them to quickly track the trend in this field. This study uses the bibliometric method to identify the evolutionary development and recent hotspots of hypertensive nephropathy. The Web of Science Core Collection database was used to extract publications on hypertensive nephropathy from January 2000 to November 2023. CiteSpace was used to capture the patterns and trends from multi-perspectives, including countries/regions, institutions, keywords, and references. In total, 557 publications on hypertensive nephropathy were eligible for inclusion. China (*n *= 208, 37.34%) was the most influential contributor among all the countries. Veterans Health Administration (*n *= 19, 3.41%) was found to be the most productive institution. Keyword bursting till now are renal fibrosis, outcomes, and mechanisms which are predicted to be the potential frontiers and hotspots in the future. The top seven references were listed, and their burst strength was shown. A comprehensive overview of the current status and research frontiers of hypertensive nephropathy has been provided through the bibliometric perspective. Recent advancements and challenges in hypertensive nephropathy have been discussed. These findings can offer informative instructions for researchers and scholars.

## Graphical abstract

**Figure:**
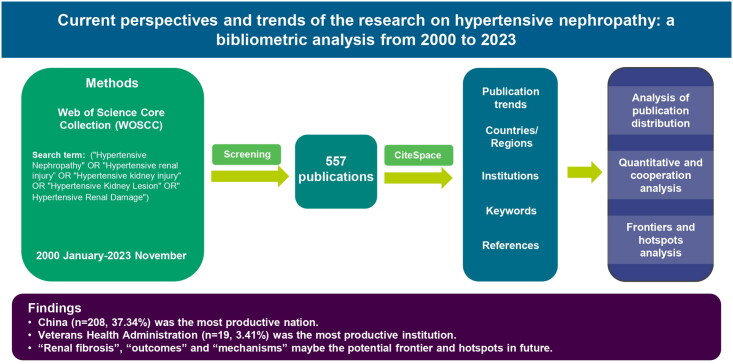

## Introduction

Hypertension is a prevalent chronic condition that is a major risk factor for ischemic heart disease, chronic kidney disease (CKD), and other vascular disorders globally [1]. Hypertensive nephropathy (HTN) is regarded as the consequence of chronic high blood pressure, which is the second leading cause of end-stage renal disease (ESRD) after diabetes mellitus [2–4]. The clinical manifestations are nocturia, proteinuria and decreased glomerular filtration rate (GFR). Most patients develop mild-to-moderate hypertensive nephrosclerosis, and only patients who suffer from chronic uncontrolled blood pressure or preexisting kidney disease, will progress to CKD and eventually end in ESRD [5]. Since the disease is related to significant morbidity and death [6], there is an urgent need to diagnose and manage it at an early stage.

Hypertension and CKD are the two main diagnostic requirements for HTN. However, in most cases, the order of these diagnostic events is hard to confirm. A large amount of CKD patients are associated with high blood pressure, and CKD patients with hypertension will fulfill the diagnosis of HTN [7]. At present, the diagnosis of HTN is still one of exclusion. The effective strategy to slow the progression of HTN is to control albuminuria and blood pressure, which can be attended to through both lifestyle interventions and pharmacological methods. The non-pharmacologic interventions include weight reduction, moderately intense physical activity, and sodium restriction [8]. While these approaches only have a complementary impact, most require medication to manage HTN. In the initial stage, renin-angiotensin system (RAS) blockers such as angiotensin-converting enzyme (ACE) inhibitors (ACEi) or angiotensin receptor blockers are recommended. Diuretics or calcium blockers are also commonly applied to control blood pressure. For patients with advanced stages, such as renal failure or ESRD, kidney replacement therapy, including dialysis or transplantation, is the common method to support and maintain the quality of life [6].

The mechanisms of HTN have been investigated for a long period and are extensively discussed in the highly cited reviews [5,9–11]. The major pathogenic events are renal hemodynamic changes and vascular remodeling. When hypertension occurs, the change in the renal hemodynamics will affect the renal arterioles. Initially, the arteriole will increase responsiveness to vasoconstrictive substances, resulting in increased vascular resistance and decreased local blood flow. If hypertension persists, structural changes will occur, leading to thickening the intima of small arterioles, narrowing the afferent arteriole, and causing hyalinosis [12,13]. These changes lead to partial glomeruli ischemia which ends with shrinkage of glomerular tufts and podocyte loss [5]. To maintain filtration, the remaining glomeruli become hypertrophic and result in microalbuminuria. Hypertension also affects the tubular cells leading to cell flattening and cell atrophy. Moreover, the mechanical stress derived from high blood pressure and stimulation of RAS triggers epithelial-mesenchymal transition (EMT), contributing to tubulointerstitial fibrosis and eventually loss of low molecular weight proteins. Therefore, hypertension-induced alterations in the vascular, glomerular, and tubular compartments are responsible for the development of CKD.

The intrarenal activation of RAS is the primary drive in hypertension-induced fibrosis [14]. The RAS has two pathways, the classical and the alternative pathway. The classical pathway started with the synthesis of renin by juxtaglomerular cells in the kidney. Then the renin acts on the angiotensinogen to produce angiotensin I (Ang I), which is transformed by the ACE into angiotensin II (Ang II), the major player in hypertension. The Ang II binds to two distinct receptors, Ang II type 1 (AT1R) and type 2 (AT2R) receptors in different tissues to exert its actions [15]. Ang II binds AT1R to exert major physiological and pathophysiological actions, such as vasoconstriction, sodium and water retention, inflammation, and fibrosis [16]. While the ATR2 signaling exerts the opposite function of those of AT1R [17]. The major function of Ang-II is to synthesize collagen and fibronectin through transforming growth factor-β (TGF-β)-independent mechanisms [18]. While Ang II is also found to stimulate the TGF-β pathway, which is involved in tissue fibrosis [19]. In the alternative pathway, the ACE2 and its product Angiotensin 1-7 binds to the Mas receptor, exerting protective effects in the fibrogenesis and inflammation in target organs, antagonizing effects against the classical pathway [20]. However, for patients with hypertensive or diabetic kidney diseases, the downregulation of ACE2 and upregulation of ACE and AT1R has been noticed [21–23]. Moreover, ACE2 deficiency is a progressive factor in renal fibrosis [24,25].

In recent years, novel mechanisms contributing to HTN have been widely investigated. The heat shock protein (Hsp) 70 chaperone has been found to have cytoprotective effects on the Ang II-induced EMT after AT1R blockage [26]. Moreover, the hemichannels (HC) and pannexons, which are critical channels for intracellular Ca^2+^ signaling, have been explored their roles in the Ang II-mediated renal damage [27]. Additionally, the abnormal activation of the complement system [28] and ferroptosis [29] also contributed to the pathogenesis of HTN.

Driven by the health burden of HTN, there is a considerable number of studies describing the advancements in the emerging mechanisms and therapeutic interventions. Moreover, the dramatic increase in publications hinders researchers from keeping up with the current trends and frontiers in this field. Therefore, it is important to comprehensively review the current perspectives and trends of HTN. Although systemic reviews and meta-analyses can provide a brief summary of research in this field, some dimensions of literature, such as time and space, are hard to present by these knowledge synthesis methods. Bibliometrics is a novel tool to apply mathematical and statistical methods to quantitatively analyze literature [30]. Historical bibliometrics, as one of the subfields of bibliometrics, provides the dimension of time and space to bibliometric analysis, allowing scientists to learn the patterns and trends of a specific specialty or field [31]. To present the information in visual maps, bibliometric analysis relies on various softwares, such as CiteSpace and VOSviewer [32,33]. Although HTN has been reviewed from multiple perspectives, there remains a lack of a holistic historical analysis to present the current views and progress in this arena. To close the gap, we used historical bibliometrics to analyze the trend in publication production, the evolution of keywords, and highly cited references. In this article, we aimed to visualize and evaluate the current status and hotspots of HTN through bibliometrics to provide useful instructions for scientists and clinicians. This study serves as a historical guide for experts and newcomers to identify the evolution of this field, target fresh topics of interest, and facilitate scholars to improve their research plans.

## Materials and methods

### Data source

**Figure 1.**
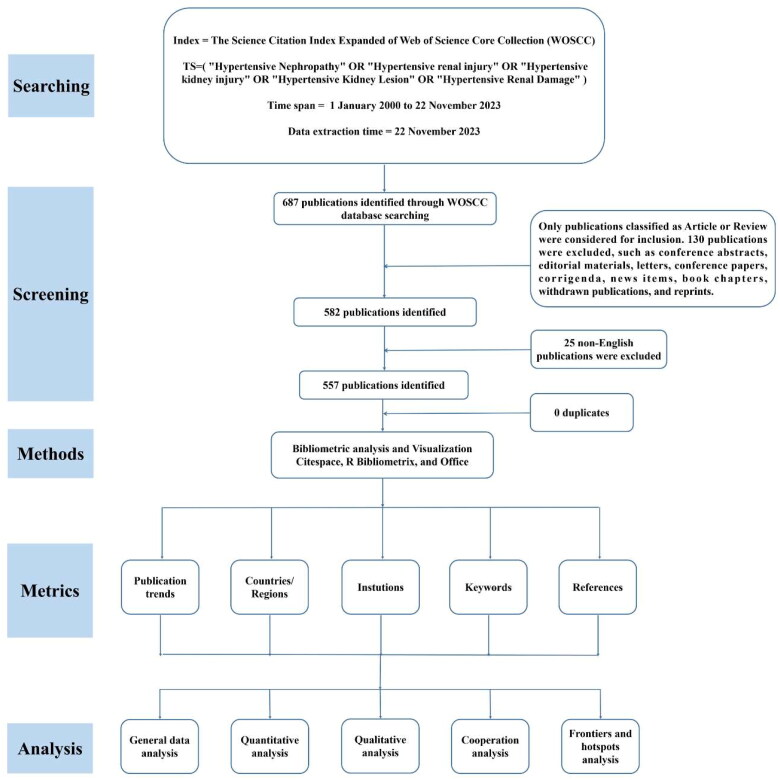
Flowchart of publication extraction and an overview of the bibliometric analyses contained in this study. To present a comprehensive overview of the evolution of hypertensive nephropathy, we collected scientific publications from 2000 to 2023 and employed a bibliometric analysis, illustrating retrieved literature from five diverse perspectives, including the publication trends, leading countries/regions, institutions, keywords and references.

The Web of Science Core Collection (WOSCC) is one of the most comprehensive and authoritative databases and is extensively applied in bibliometric analysis [34–36]. The data file collected from WOSCC could be directly imputed into the bibliometric software CiteSpace. The procedures for data retrieval and bibliometric analysis have been displayed in Figure 1.

### Search strategy

The Science Citation lndex (SCI) Expanded of the WOSCC database was used to extract documents published from January 2000 to November 2023. All publications were downloaded on November 22 2023 to minimize bias due to database updates. We finally use the search terms: TS= (‘Hypertensive Nephropathy’ OR ‘Hypertensive renal injury’ OR ‘Hypertensive kidney injury’ OR ‘Hypertensive Kidney Lesion’ OR ‘Hypertensive Renal Damage’). Moreover, only articles and reviews written in English were included. In the end, there were 557 articles left. The literature retrieved from WOSCC was converted to plain text format for export, which was named ‘download_XXX.txt’, including complete records and references. These data were imported into CiteSpace for visualization and analyzation, and Microsoft Excel for Tables.

### Bibliometric analysis

CiteSpace, a Java application, is developed by Dr. Chaomei Chen, which allows the detection and visualization of trends and patterns in publications obtained from bibliometric databases [37]. CiteSpace also produces various metrics to present the knowledge discovery, including structural metrics (e.g., centrality, modularity, silhouette), and temporal metrics (e.g., citation burstness), etc. The centrality reflects the significance of the subject, and the node with high centrality is an indicator of key hubs. The hub is highlighted with a purple ring to stand out in the network. The modularity (*Q*-score) of a network reveals the degree the network can be partitioned into clusters and the silhouette (*S*-score) reflects data consistency within clusters [38]. The cluster is regarded as important if the *Q*-score is more than 0.3, and a well-structured network is indicated by a larger value. The network is considered homogenous and reliable when the *S*-score is over 0.7 [39]. However, an *S*-score of 1 may reflect the relevant cluster is relatively isolated. The burst detection is available in CiteSpace and is applied to indicate a sharp increase of interest in a speciality [40].

For this paper, we collected the following significant data: the countries/regions, institutions, keywords, and references. The output of the bibliometric analysis was presented as visual networks generated by CiteSpace (CiteSpace 6.2.R5, advanced) and R Bibliometrix. Tables were constructed by Microsoft Excel. More instructions about the bibliometric analysis can be found in this paper [41].

## Results

### Publication trends

**Figure 2.**
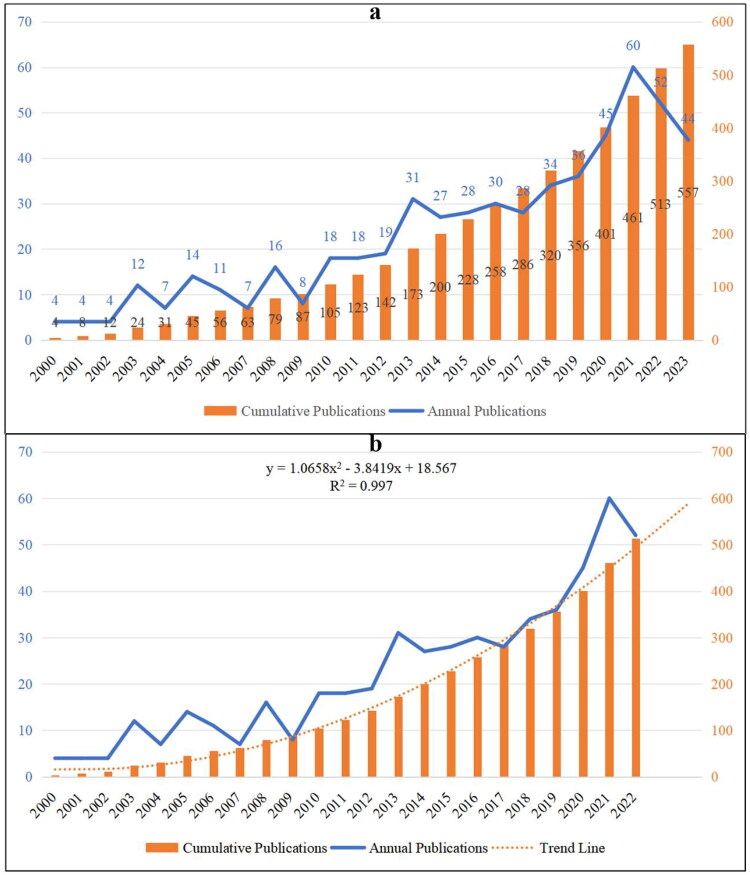
Counts and trends related to the publications of hypertensive nephropathy. (a)The annual quantity and trend of publications from 2000 to 2023. (b) The expected trendline of cumulative publications based on the relevant linear calculation. The trendline is in accordance with a second-order polynomial distribution.

In total, 557 publications related to HTN were retrieved from the WOSCC databases without duplicates. The annual and cumulative output of HTN from 2000 to 2023 is displayed in Figure 2. In general, the number of global articles on HTN increases with fluctuations, rising from 4 in 2000 to 557 in 2023, with a 2.40% annual growth rate (Figure 2a).

Since the data collection ended in November 2023, this year was excluded from the relevant linear calculation. As shown in Figure 2b, there is an incremental trendline regarding the number of cumulative publications from 2000 to 2022 (*R*^2^ = 0.997). The expected trendline is under a second-order polynomial distribution, and the cumulative counts of publications in this field is expected to be around 600 from 2000 to 2023.

### Countries/regions

**Figure 3.**
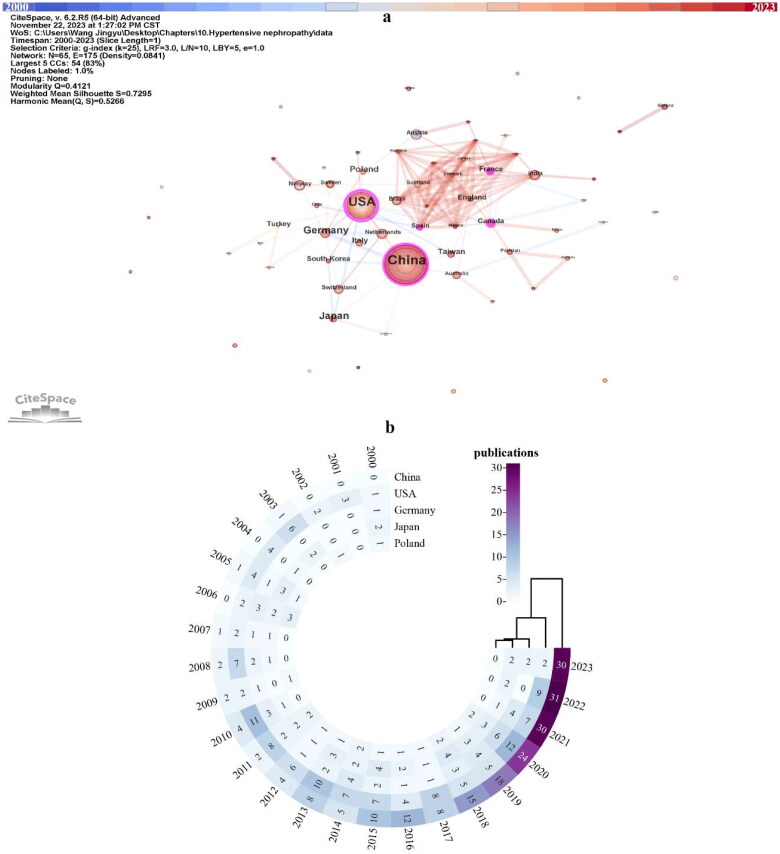
Countries contributed to the research of hypertensive nephropathy. (a) Visualized network of all 65 countries/regions, 2000–2023. (b) Circo heatmap displaying annual publications of top five most prolific countries/regions, 2000–2023.

In total, there are 65 countries/regions contributed to the research on HTN. According to Figure 3 and Table 1, China (*n* = 208, 37.34%) is the most productive country in the research of HTN, followed by the USA (*n* = 134, 24.06%), Germany (*n* = 44, 7.90%), Japan (*n* = 43, 7.72%) and Poland (*n* = 21, 3.77%). The USA continually devoted to this field since 2000, and steadily published over four articles annually from 2010 to 2022 (Figure 3b). Chinese scholars started later compared with the USA, while the publications in China surged in 2013 and kept more than eight annual outputs since 2015 (Figure 3b). Of note, since 2021, the publications from China are steadily at 30, indicate the fact that Chinese researchers pay huge attention in HTN in recent years.

**Table 1.** The top five most productive countries/regions in the research of hypertensive nephropathy.

Ranking	Country/Region	Count	Centrality	H-index	AC/P
1	China	208	0.12	32	19.45
2	USA	134	0.45	47	52.07
3	Germany	44	0.03	18	32.25
4	Japan	43	0.01	18	24.81
5	Poland	21	0.01	13	20.43

AC/P: average citations/per publication.

Centrality in the CiteSpace is a measure of the importance of network nodes [42]. A node with more than 0.1 centrality, is recognized with a relatively great influence in this field and is highlighted with purple in the visualizations. The centrality of the country can reflect the international recognition of that country in a specific field. The USA has the highest degree of centrality (0.45) among countries and regions, indicating its robust communications with other countries in the research of HTN (Figure 3a, Table 1). Despite the number of publications, Canada, France, and Spain have over 0.1 centrality. The close cooperation between these three countries and other nations could also be noticed in Figure 3a. The average citations/per publication (AC/P) can partially explain the attraction of the literature. The AC/P in the USA (*n* = 52.07) is the highest, followed by Germany (*n* = 32.25), Japan (*n* = 24.81), and Poland (*n* = 20.43). The h-index is commonly recognized as an indicator of academic output, which can be used to evaluate the quality and quantity of research in a country, a journal, or an institution [43,44]. Except the USA has the highest h-index (47), the ranking of the h-index is generally parallel to the number of publications (Table 1).

### Institutions

**Figure 4.**
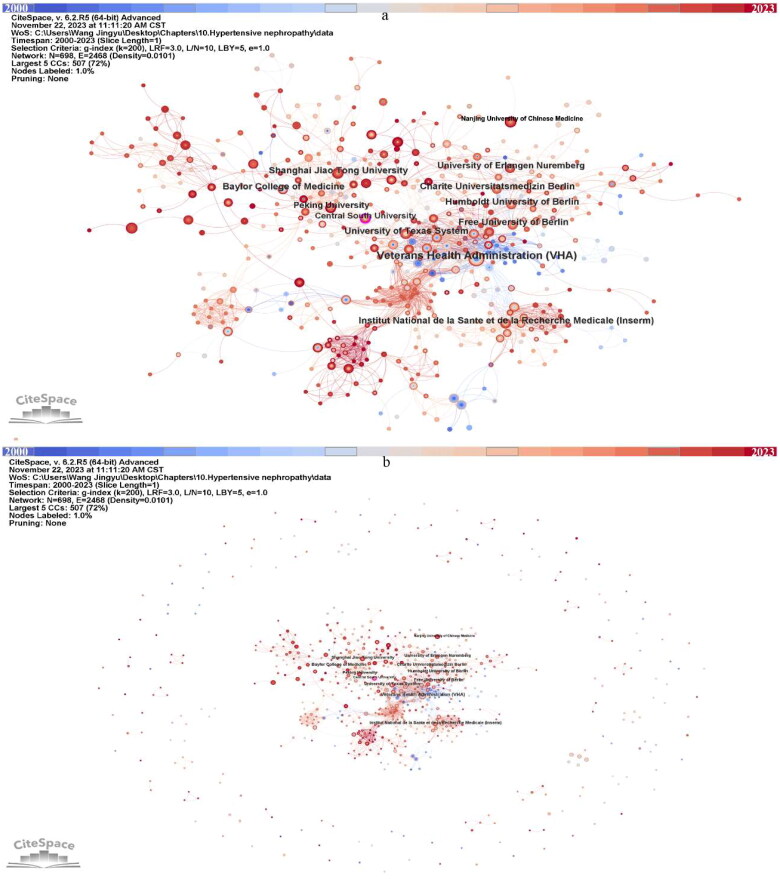
Visualized network displaying the collaborative relationships among 698 institutions displayed in CiteSpace, 2000–2023. (a) zoom in presentation of the key institutions. (b) overview of all the institutions.

Over 1000 institutions participated in the study of HTN, we extracted 698 institutions centered in the CiteSpace visualization for further analysis (Figure 4). We can notice extensive cooperation between institutions (Figure 4a). Institutions in the United States, Germany, China, and France occupy the top list regarding publications (Table 2). Veterans Health Administration (VHA) ranked first (*n* = 19) and has 0.10 centrality and extensive cooperation with other institutions could be noticed. Although the other institutions devoted more than 10 publications in this field, their centrality cannot attach 0.1 (Table 2). It is noteworthy that Central South University has over 0.1 centrality in the institutional visualization (Figure 4a). The VHA (*n* = 73.42) and Baylor College of Medicine (*n* = 69.00) have very high AC/P, followed by Institut National de la Sante et de la Recherche Medicale (Inserm) (*n* = 29.09) and University of Erlangen Nuremberg (*n* = 27.00). The ranking of the h-index follows the ranking of publication amount.

**Table 2.** The top eleven most productive institutions in the research of hypertensive nephropathy.

Ranking	Institution	Count	Centrality	H-index	AC/P
1	Veterans Health Administration (VHA)	19	0.10	14	73.42
2	Baylor College of Medicine	12	0.07	9	69.00
3	Charite Universitatsmedizin Berlin	12	0.02	8	17.92
4	Free University of Berlin	12	0.02	8	17.92
5	Humboldt University of Berlin	12	0.02	8	17.92
6	Shanghai Jiao Tong University	12	0.05	7	19.58
7	Institut National de la Sante et de la Recherche Medicale (Inserm)	11	0.08	7	29.09
8	University of Texas System	11	0.09	8	23.27
9	Peking University	10	0.06	6	20.70
10	University of Erlangen Nuremberg	10	0	6	27.00
11	Nanjing University of Chinese Medicine	10	0	6	9.50

AC/P: average citations/per publication.

### Keywords

**Figure 5.**
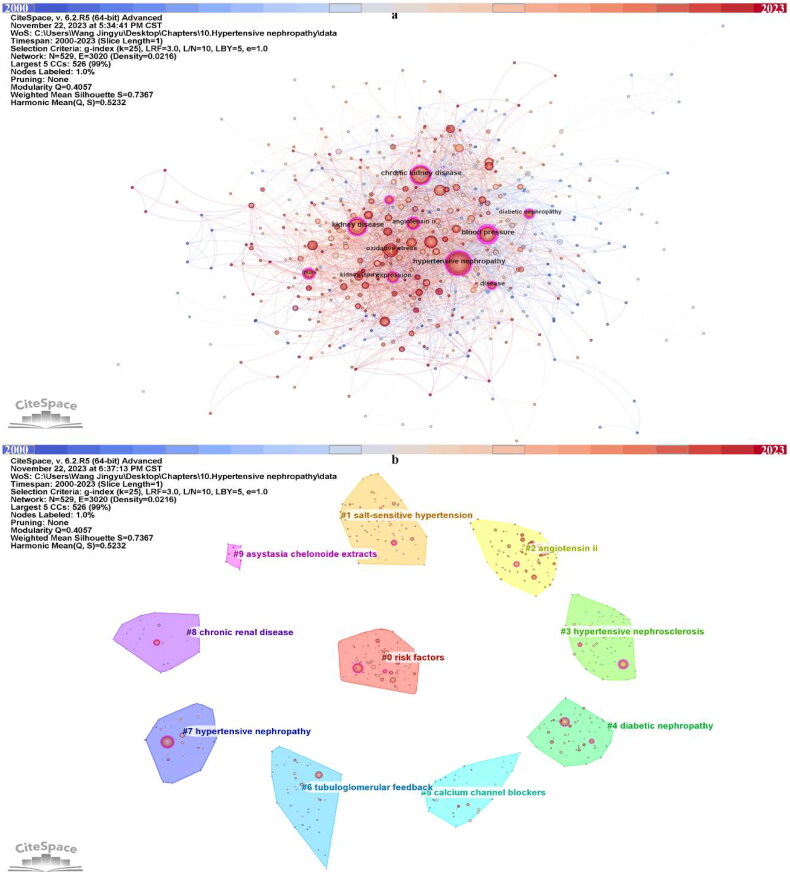
Keywords in the publications of hypertensive nephropathy. (a) Keyword visualization. (b) Keyword clustering.

A total of 529 keyword nodes were visualized in the maps after merging synonyms and eliminating nonsense terms (Figure 5a). Each nodes represent a keyword, and the size of nodes can reflect the frequency of this keyword appears in this field. Labels were given to the top ten most used keywords. ‘Hypertensive nephropathy’ appeared 139 times and ranked first, followed by ‘chronic kidney disease’ (123), ‘blood pressure’ (98), ‘kidney disease’ (97) and so on (Table 3). Among the top ten most common keywords, eight of them have over 0.1 centrality, and only ‘oxidative stress’ and ‘kidney injury’ has less than 0.1 centrality (Table 3). Moreover, most of them were used in a quite early timepoint, from 2000 to 2003.

**Table 3.** The top ten most used keywords in the research of hypertensive nephropathy.

Ranking	Keyword	Count	Centrality	Year
1	Hypertensive nephropathy	139	0.15	2002
2	Chronic kidney disease	123	0.18	2003
3	Blood pressure	98	0.28	2002
4	Kidney disease	97	0.14	2003
5	Angiotensin ii	74	0.12	2000
6	Expression	74	0.14	2000
7	Disease	68	0.14	2001
8	Oxidative stress	59	0.07	2011
9	Kidney injury	53	0.08	2002
10	Diabetic nephropathy	46	0.11	2000

**Figure 6.**
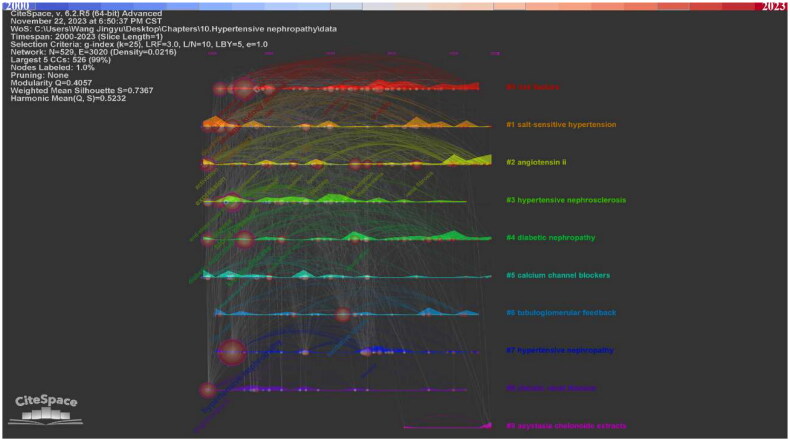
The keyword timeline and ridge plot for keyword clusters.

In addition, we clustered the keywords into ten clusters. In CiteSpace, the* Q*-score and *S*-score are parameters to determine the visualization network and clustering. In the clustering map generated by CiteSpace, the *Q*-score is 0.4057 and the *S*-score is 0.7367, indicating the division is reasonable (Figure 5b, Table 4). Compared with nodes in other clusters, nodes within the same cluster may have a comparable direction. The ten clusters are #0 risk factors, #1 salt-sensitive hypertension, #2 angiotensin ii, #3 hypertensive nephrosclerosis, #4 diabetic nephropathy, #5 calcium channel blockers, #6 tubuloglomerular feedback, #7 hypertensive nephropathy, #8 chronic renal disease, #9 asystasia chelonoide extracts. The keyword timeline and ridge plot for the keyword clusters have been presented to track the developmental trend of the ten clusters (Figure 6). Of notice, cluster #1 salt-sensitive hypertension, #2 angiotensin ii, #4 diabetic nephropathy and #5 calcium channel blockers appear throughout the whole timeline from 2000 to 2023. #0 risk factors, #3 hypertensive nephrosclerosis, #6 tubuloglomerular feedback, #7 hypertensive nephropathy, #8 chronic renal disease appeared early while ended in recent years. In contrast, the #9 asystasia chelonoide extracts have emerged in recent years.

**Table 4.** Keyword clustering.

Cluster ID	Label	Size	Silhouette	Mean (year)
0	Risk factors	89	0.665	2014
1	Salt-sensitive hypertension	75	0.733	2010
2	Angiotensin ii	69	0.716	2014
3	Hypertensive nephrosclerosis	68	0.765	2008
4	Diabetic nephropathy	61	0.709	2014
5	Calcium channel blockers	42	0.86	2006
6	Tubuloglomerular feedback	41	0.749	2011
7	Hypertensive nephropathy	37	0.727	2012
8	Chronic renal disease	28	0.754	2007
9	Asystasia chelonoide extracts	7	0.981	2022

**Figure 7.**
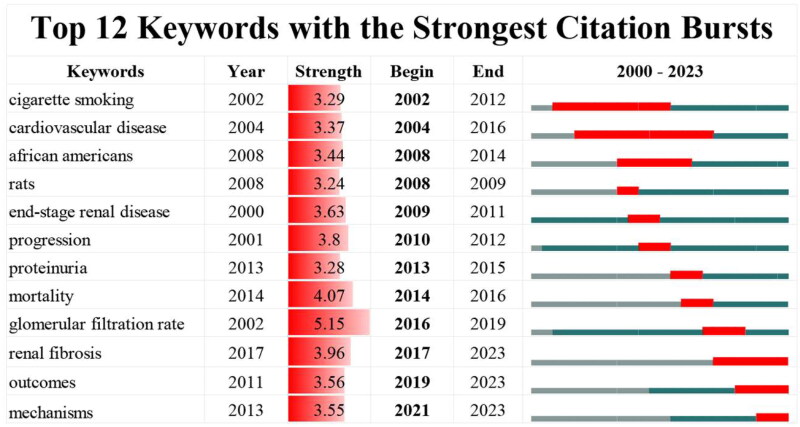
The top twelve keywords with the strongest citation bursts in the field of hypertensive nephropathy.

The emerging trend in the research area can be predicted using burst keywords. The top twelve keywords with the strongest burst strength have been presented in Figure 7. The strength of these keywords is generally distributed in a similar manner, ranging from 3.24 to 5.15. The ‘glomerular filtration rate’ has the highest strength (strength = 5.15). ‘Cigarette smoking’, ‘cardiovascular disease’ and ‘african americans’ are three keywords that come out in the early stage of the research period and burst for a relatively long period. The other keywords burst have shorter lastingness. It is worth mentioning that ‘renal fibrosis’, ‘outcomes’ and ‘mechanisms’ consistently burst into 2023.

### References

**Figure 8.**
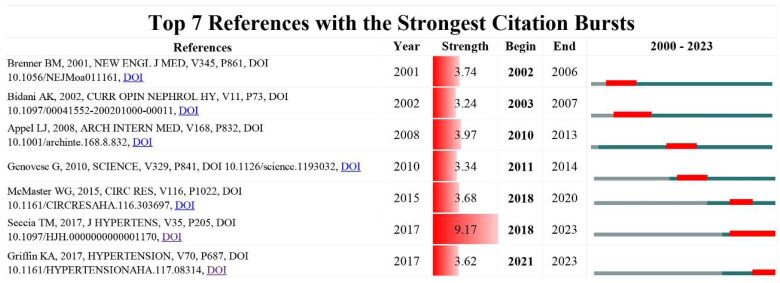
The top seven references with the strongest citation bursts in the field of hypertensive nephropathy.

The top seven references with the strongest citation bursts are shown in Figure 8 and Table 5. The burst strength of the top seven references ranged from 3.24 to 9.17. Most studies only burst for less than four years. The review titled ‘Hypertensive nephropathy. Moving from classic to emerging pathogenetic mechanisms’ published in 2017 ranked first with a total citation of 42 (strength = 9.17) and is burst to 2023. Another study burst to 2023 is the review titled ‘Hypertensive Kidney Injury and the Progression of Chronic Kidney Disease’ (strength = 3.62).

**Table 5.** The top seven references with the strongest bursts of citations in the research of hypertensive nephropathy.

Ranking	Title	Publication type	Primary research work	PMID
1	Effects of losartan on renal and cardiovascular outcomes in patients with type 2 diabetes and nephropathy	Clinical trial	Angiotensin-II–receptor antagonist losartan has achieved significant renal benefits in patients with type 2 diabetes and nephropathy, and no severe adverse effects were detected.	11565518
2	Long-term renal consequences of hypertension for normal and diseased kidneys	Review	This review systemically discusses the pathogenesis of hypertensive renal injury and impact of antihypertensives in renal damage.	11753090
3	Long-term effects of renin-angiotensin system-blocking therapy and a low blood pressure goal on progression of hypertensive chronic kidney disease in African Americans	Article	This study investigates the long-term effects of antihypertensive drugs in 1094 African Americans with hypertensive chronic kidney disease (CKD). Despite the benefit of therapy, most African Americans with hypertensive CKD continue to progress in the long-term follow up.	18443258
4	Association of trypanolytic ApoL1 variants with kidney disease in African Americans	Article	In this study, two trypanolytic APOL1 variants have been founded in African Americans, while these two variants were absent in European origin. The African specific variants may contribute to the higher rates of renal disease in African Americans.	20647424
5	Inflammation, immunity, and hypertensive end-organ damage	Review	This paper demonstrates the role of oxidative stress and immune activation in hypertension, revealing how the innate and adaptive immune systems participate in hypertension and end-organ damage.	25767287
6	Hypertensive nephropathy. Moving from classic to emerging pathogenetic mechanisms	Review	This article focuses on the novel mechanisms underlying renal damage in hypertensive kidney disease and discusses the therapeutic implications of viable podocytes or podocyte-derived proteins.	27782909
7	Hypertensive Kidney Injury and the Progression of Chronic Kidney Disease	Review	This study provides an overview of the use experimental models to elucidate the unresolved mechanisms of hypertensive renal damage.	28760941

## Discussion

### Analysis of publication distribution

As shown in Figure 2, the global publications related to HTN showed an incremental trend, from 4 publications in 2000 to 557 publications in 2023. Noticeably, there are five inflection points regarding the annual publication, which are the year 2003, 2005, 2008, 2013, and 2021, indicating HTN have attracted more attention in these five years. In particular, the annual publication in 2021 (*n* = 60) is the highest from 2000 to 2023, which accounted for 10.77% of the total quantity. Meanwhile, the relevant linear calculation indicates a stable incremental trend regarding the total number of publications (y = 1.0658x^2^ − 3.8419x + 18.567, *R*^2^ = 0.997). Therefore, this field has good research and production sustainability, more advancements will be achieved in this field.

### Countries/regions and institutions

China is the most productive nation in the field of HTN from 2000 to 2023. However, the quality of publications in China requires to be improved compared with other productive countries. These may be attributed to the imbalanced academic capabilities among institutions and the unsound academic systems [36]. Moreover, economic strength is a key determinant of scientific productivity. Only two of the top five most productive countries are East Asian, with the majority being Western European and North American nations. China is the sole developing nation, and the left all belongs to developed countries. These may indicate that high-income nations are more prone to have the burden of HTN and make more effort in this area. With the advance of globalization, international and inter-organizational collaboration becomes a good strategy to elevate the quality and quantity of research. Although experts from Europe and North America do not publish much work annually, their status is centralized in the visualized network among global countries. This may be associated with their robust international cooperation with other productive countries. Institutions from Germany are highly active in the research of HTN. Of note, VHA is the leading institution in this field worldwide. Less communication in Asian countries and institutions was evident. Therefore, it is critical to promote international cooperation and communication to raise the caliber and quantity of publications in these regions.

### References

The reference with a strong citation burst is an indicator of the research interest in a given period. Among the top seven references with the strongest citation bursts, two are clinical trials investigating the effects of antihypertensive drugs, including Ang II-receptor blockers in patients with renal disease [45,46]. Another article describes the novel trypanolytic apolipoprotein L-1 (APOL1) variants specifically expressed in African Americans may contribute to the increased rate of renal disease [47]. The others are reviews describing the mechanisms of HTN from different aspects [5,9–11].

In the early stage of research in HTN, most studies are investigating the effect of anti-hypertensive drugs on renal function. Patients with diabetes nephropathy often suffer from the progressive decline of renal function. The hypertensive treatment can achieve renoprotective effects. The ACEi have been investigated for their effects in patients with type 1 [48] and type 2 [45] diabetes. The ACEi Captopril has been shown to protect insulin-dependent diabetic nephropathy from deterioration in renal function [48]. The Ang II-receptor antagonist losartan, alone or combined with conventional antihypertensives, was well tolerated and achieved significant renal benefits in patients with type 2 diabetes and nephropathy [45]. The ACEi Benazepril has been validated its ability to protect from renal insufficiency in patients with different renal diseases [49]. Another ACEi ramipril has been shown to safely reduce proteinuria and the rate of GFR decline in non-diabetic renal diseases [50]. The posttrial follow-up study showed that ramipril seemed to stabilize the GFR and prevent ESRD for up to 54 months [51].

A large proportion of African Americans with ESRD are attributed to hypertension, and the treatment response to RAS-blocking therapy is different compared with Americans of white origin. Ramipril has been reported with superior performance in the reduction of GFR, ESRD, or death in African Americans with hypertensive CKD, compared with calcium channel blocker amlodipine and β-blocker metoprolol [52–54]. The follow-up study [46] further evaluated the long-term effects of three antihypertensive therapies, ACEi, calcium channel blocker, and β-blocker. Most African Americans with hypertensive CKD continued to progress despite the use of ACEi therapy and the achievement of recommended blood pressure goals. While this trial only enrolled African Americans, it is unable to speculate the response of antihypertensive therapy in different races. However, an early clinical trial [55] has investigated the effect of blood pressure control on renal function in black and white populations with mild to moderate hypertension. The results show that blood pressure control has been shown to important in renal function maintenance in white men but not blacks.

Due to the ethnic variation in hypertensive renal disease, more studies are investigating the unknown mechanisms underlying the poor response in African ancestry. The nonmuscle myosin heavy chain 9 (MYH9) is strongly associated with hypertension-associated ESRD in African Americans [56]. Two distinct alleles encoding APOL1, a high-density lipoprotein against Trypanosoma brucei parasite, have been found to be exclusively expressed in African chromosomes while not in Europeans, which could account for the increased CKD rate in the African population [47]. The APOL1 risk variants are highly expressed in African origin, while virtually absent in European and Asian origin [57]. Interestingly, the APOL1 variants have also been found to influence renal transplantation. The kidney donors with two APOL1 risk variants have been related to shorter graft survival and higher HLA mismatch, while not for African ancestry excluding APOL1 [58]. Therefore, the poor treatment response may relate to the genomic insults in this population.

Apart from the research investigating the genetic risk factors, mechanisms underlying HTN have been widely discussed [5,9–11]. The review published in 2002 discussed the pathophysiology of hypertensive renal damage in animal models, antihypertensives, genetic factors, and results from human studies [11]. After decades of efforts, more studies have contributed to the pathogenetic mechanisms underlying hypertension-induced renal damage. The rest reviews based on the available research results, have elucidated the mechanisms from different aspects. One study examined the processes by which the innate and adaptive immune systems contribute to renal and vascular dysfunction, raising blood pressure and causing end-organ damage [10]. While this study burst in 2018 and ended in 2020. The other two reviews published in 2017 have received recent attention and have burst to 2023, indicating their lasting influences. The study ‘Hypertensive nephropathy. Moving from classic to emerging pathogenetic mechanisms’ has received recent attention from 2018 to 2023. It describes novel mechanisms, including podocyte loss and EMT, underlying the renal damage in hypertension and the associated therapeutic implications [5]. Another study ‘Hypertensive Kidney Injury and the Progression of Chronic Kidney Disease’ burst from 2021 to 2023 [9]. This review mainly stated the experimental views of hypertensive renal injury derived from animal models and the implications in clinical settings. While the study from Seccia et al. has the strongest strength (9.17) among the references, suggesting the perspectives in this review have been widely adopted and explored in recent research.

### Keywords

A keyword with high burst strength is a roadmap of the hotspots of research frontiers over time. Of note, ‘cardiovascular disease’ and ‘african americans’ are two keywords that have been highly mentioned in the early stage of this field (Figure 7), corresponding with the above references analysis. The keywords ‘end-stage renal disease’, ‘proteinuria’, ‘morality’, and ‘glomerular filtration rate’ were widely involved in the outcome analysis of HTN.

According to Figure 7, the keyword ‘renal fibrosis’, ‘outcomes’ and ‘mechanisms’ citation burst continues till now, suggesting that these subjects has drawn interest consistently and may represent the frontiers in this field.

### Mechanisms in hypertensive nephropathy

Since the RAS plays a key role in hypertension and related kidney damage, Ang II, the most powerful vasoconstrictor of the RAS, has been extensively explored in the pathogenesis of HTN. Ang II can upregulate the ACE expression, while downregulate Ang II-breakdown enzyme (ACE2) expression through activation of extracellular signal-regulated kinase (ERK)1/2 and p38 mitogen-activated protein (MAP) kinases, leading to ACE/ACE2 imbalance and contributing to hypertensive-induced renal damage [23]. Ginsenoside Rg3 attenuated the Ang II-mediated renal damage by upregulating tissue ACE2 in spontaneously hypertensive rats [59]. Naringenin has been found to retard the rise of Ang II levels and inhibit the increase of ACE/ACE2 and AT1R/AT2R protein ratio in mice models [60]. The opening of hemichannels and pannexons depended on intracellular Ca^2+^ signaling, while the Ang II-mediated dysfunction of Ca^2+^ signaling, led to renal damage [27]. Therefore, the hemichannels and pannexons may be novel targets in the treatment of Ang II-induced renal damage.

In patients with hypertension, the sympathetic adrenaline system is hyperactivated, leading to renal arteriole contraction and vascular resistance, affecting vascular remodeling. The micro-RNA has been found to contribute to nerve activity. The miR-22 expression is elevated in spontaneously hypertensive rats, reducing the expression of chromogranin A (CHGA), resulting in higher central and peripheral nerve activity, contributing to the pathogenesis of hypertension [61]. Genetic variation in the CHGA 3′-region has been shown to promote the inhibition of CHGA by miR-107, leading to increased blood pressure and contributing to hypertensive renal damage [62,63]. Moreover, QiShenYiQi, a traditional Chinese medicine for cardiovascular disease, has been found to alleviate renal damage through downregulating alpha-1D adrenergic receptors and upregulating salt-inducible kinase 1 in the mice model [64].

Oxidative stress and immune activation have been shown to play a key role in HTN, which is mainly signaling through TGFβ/Smad and NF-κB (nuclear factor κB) pathways [10]. Smad7 as a downstream inhibitor of both pathways [65], could block Ang II-induced hypertensive renal disease in mice [66]. The overexpression of smad7 also prevented Ang II-induced loss of renal miR-29b, an miRNA that inhibits both TGFβ/Smad3 and NF-κB pathways. However, the loss of ACE2 promotes Ang II-induced renal damage by targeting smad7 for ubiquitin degradation. Overexpression of renal smad7 could protect against renal injury through dual-inhibition of TGF-β/Smad3 and NF-κB signaling and targeting the Smad3-dependent miR-21 axis [67]. Other novel mechanisms underlying oxidative stress and inflammation have also been investigated in the field of HTN. Hydrogen sulfide has been found to reduce oxidative stress and renal inflammation, rendering it a potential therapeutic target in hypertensive-related renal damage [68]. Hyperhomocysteinemia (HHcy) appears to synergistically aggravate hypertension-related vascular injury by enhancing oxidative stress [69]. Furthermore, HHcy stimulates B cells to secrete pathogenic anti-beta 2 glycoprotein I antibodies exacerbating hypertensive kidney damage by inducing glomerular endothelial cells ferroptosis [70]. Therefore, targeting B cells or ferroptosis may be promising strategies for treating HHcy patients with renal injury. Serum interleukin-22 (IL-22) and Th22 cells have been found to be enhanced in patients with hypertensive renal damage. AT-1 blocker irbesartan has been found to ameliorate inflammation and fibrosis in Ang II-induced renal damage via inhibiting Th22 cell chemotaxis and infiltration [71]. The anti-IL-22 could reduce inflammation and renal fibrosis in Ang II-treated mice [72].

To date, multiple studies focus on traditional Chinese herbs, and many of them are found to alleviate inflammation and oxidative stress in HTN. Qian Yang Yu Yin Granule has been found to relieve hypertensive renal injury through multiple mechanisms, including a nicotinamide adenine dinucleotide phosphate (NADPH)-oxidase (NOX)-reactive oxygen species (ROS) pathway [73], a novel epigenetic mechanism related to Nicotinamide N-Methyltransferase expression, and reversing the Ang II-induced autophagy of podocytes [74]. Qingda granule [75] has been shown to improve renal function through inhibition of NF-κB signaling pathway and inflammation. Tengdan capsule (TDC) [76] could effectively lower blood pressure and protect against renal damage by regulating the TGF-β/Smad signaling pathway. Isoliquiritigenin can attenuate hypertensive renal injury by suppressing Ang II-induced apoptosis inflammation and extracellular matrix deposition [77]. More information about Chinese herb related mechanisms in HTN is discussed in another review [78].

Podocyte injury, EMT and tubulointerstitial fibrosis are important pathological processes in HTN [5,79]. Sirt6, a histone deacetylase involved in DNA damage repair, plays a key role in Ang II-induced DNA damage and podocyte injury [80]. A novel mechanism of silent mating type information regulation 2 homolog-2 (SIRT2)-septin 4 deacetylation axis has been found to participate in the apoptosis of podocytes, rendering it a potential pathway to treat HTN [81].

With the development of sequencing techniques and bioinformatics, more mechanisms underlying HTN have been disclosed. The novel pivotal genes have been initially identified through microarray data using bioinformatic analysis [82]. Ferroptosis has been found to play a role in HTN through pathways such as branched-chain amino acid metabolism and retinol metabolism, and biological processes such as organic and amino acid metabolism and humoral immunity [29]. The role of gastrointestinal microbiota also has been investigated through sequencing and bioinformatic analysis. The difference in the combination of *O. formigenes* and *V. parvula* is related to bile-acid metabolism in hypertensive patients and may be the factor causing CKD [83].

### Renal fibrosis in hypertensive nephropathy

Renal fibrosis is an important step in the progression of hypertension-induced renal damage. When the kidney injury happens, the local fibroblasts, pericytes, and immune cells are activated and secrete inflammatory factors, synthesizing extracellular matrix (ECM) proteins to participate in the wound healing process. If the process is deregulated, excessive deposition of ECM components in the kidneys will lead to tissue disruption and renal dysfunction [84,85]. The activation of myofibroblasts and deposition of ECM are the main events in renal fibrosis. When the repetitive injury happens in the tubular epithelium, the epithelial cells fail to re-differentiate, resulting in epithelial dedifferentiation, and activation of the Notch [86], Wnt [87], Hedgehog (Hh) [88], and SRY-related high-mobility-group box 9 pathways [89]. The signaling changes in the injured tubules initiate the secretion of paracrine factors such as TGF-β, Wnt, and Hh ligands, affecting the interstitial fibroblasts and pericytes nearby to stimulate myofibroblasts and ECM deposition [90]. Meanwhile, various immune cells, including macrophages [91], lymphocytes [92], neutrophils [93], and other immune cells [94] are found in the fibrotic niche and contribute to the scaring process via molecular factors. Furthermore, emerging research suggests that epigenetic processes such as DNA methylation, chromatin histone modifications, and non-coding RNAs may be responsible for the progression of kidney disease. These epigenetic pathways could also be useful in the development of promising translational biomarkers and therapeutic targets [95,96].

Since renal fibrosis remains a key determinant in hypertensive renal damage, it is important to diagnose and manage the fibrosis process in the early stage. Albuminuria and GFR are ineffective at determining the extent of fibrosis; in fact, biopsy procedures remain the gold standard with invasive limitations. Hence, other noninvasive markers are required. The microRNAs such as urinary miR-21 [97] and miR-29a [98] have been identified for their potential role in the noninvasive detection of hypertensive renal injury, but whether these promising markers can achieve the same or better performance of renal biopsy still requires further validation. Further, there are no effective medications for renal fibrosis. The RAS blocker [99,100], Dapagliflozin (sodium-glucose cotransporter 2 inhibitor) [101], Atrasentan (endothelin-1 blocker) [102], Tolvaptan (vasopressin receptor 2 antagonist) [103], Finerenone (non-steroidal anti-mineralocorticoid) [104] are found to delay the progression of CKD. While these drugs are not commonly applied to renal fibrosis, more reliable therapeutic options are required in HTN. Some traditional Chinese medicine, such as TDC [76] and Bu-Shen-Jiang-Ya decoction [105] has been shown to attenuate renal fibrosis through different mechanisms. Moreover, other non-pharmacological management has been examined for clinical benefit. Daily low-intensity pulsed ultrasound therapy [106] and exercise training [107] have been found to ameliorate renal fibrosis, providing new options in the management of the disease.

### Outcomes in hypertensive nephropathy patients

To investigate the outcomes of HTN, we searched for clinical research in the ClinicalTrials.gov platform to find the associated events. We searched the ClinicalTrials.gov for phase 1–4 trials using the search term ‘hypertensive nephropathy’ on 16 December 2023. After carefully checking the research details and filtering trials unrelated to hypertensive nephropathy, only trials within phase 1 to 4 were under analysis. Phase 1–4 clinical trials involving patients with hypertensive nephropathy has been displayed in the Supplementary Table 1. While most clinical trials have no available results, only some of them documented final outcomes.

Among the study design, most trials aimed to investigate the therapeutic outcomes of interventions in HTN, and only one trial explored the preventive action of exercise programs in patients with hypertensive CKD (NCT01155128). The combined aerobic and resistance training could reduce inflammation and insulin resistance in hypertensive patients in the early stage of CKD, with no significant effect on kidney disease progression [108].

Other studies focused on the therapeutic outcomes of HTN. The significant findings are still focused on the clinical outcomes of antihypertensives in African Americans. Antihypertensives have been shown to have no significant attenuation in the progression of kidney disease [46]. For African Americans with hypertensive chronic kidney disease, both the PM (bedtime) dosing of once-daily antihypertensive or the addition of drugs taken at bedtime cannot significantly reduce nocturnal blood pressure [109]. Moreover, metoprolol has been found to increase the risk of uric acid and gout-related medication use in African American adults [110]. Other therapeutic managements also have been investigated in HTN. In hypertensive CKD patients, 6 weeks of aliskiren treatment lowers blood pressure and sympathetic activity [111]. The add-on direct renin inhibitor aliskiren safely reduced proteinuria and attenuated the decline in GFR especially prominent in patients with HTN who were receiving ARBs [112]. Moreover, the LCZ696 is a first-in-class angiotensin receptor neprilysin inhibitor, reported with safety and efficacy in blood pressure reduction in Japanese patients with hypertension and renal dysfunction without renal function decline [113]. Similar efficacy and tolerability in the therapeutic combination of azilsartan medoxomil/chlorthalidone (AZL-M/CLD) and olmesartan/hydrochlorothiazide were documented in the hypertensive participants with stage 3 CKD [114]. Since most trials have not uploaded their results, there is an urgent requirement for available clinical outcomes to fulfill the outcomes for HTN.

### Limitations

Several limitations can be noticed in this study. Firstly, the data was gathered solely from WOSCC, resulting in a relative scarcity of related papers. Despite this, WOSCC remains the most often utilized database in bibliometric analysis. Secondly, we only chose English publications, the contributions of non-English publications are underestimated. Thirdly, since the literature in WOSCC is constantly updated, the role of recently released research is undervalued because it may not be sufficiently cited. Thus, there will always be a difference between retrieved knowledge and the real-time situation. Since the quality of articles retrieved in the CiteSpace varied, the validity of the analysis may be weakened. A quality screening system in the bibliometric platforms is required to cope with the variable quality. In this study, we only used CiteSpace for the bibliometric analysis, other bibliometric knowledge has been undervalued, such as visualizations from VOSviewer. Finally, due to the lack of synthetic knowledge synthesis for the bibliometric theme analysis, more detailed instruction about the production of synthetic knowledge could be referred to in another bibliometric research [115].

## Conclusions

This is the first bibliometric analysis of HTN that we are aware of. The bibliometric study provides an extensive evaluation of the present status and development patterns in this area. Publications in HTN display an incremental trend over the past two decades, highlighted by the significant contributions of China, the USA, and Germany. The research hotspots and latest trends are believed to be the novel mechanisms related to the pathogenesis, especially the process of renal fibrosis. Additionally, clinical investigations of variable outcomes in HTN also served as the focus of future research. This study could support scientists in understanding the evolution and advancement of HTN, benefit the clinicians to cooperate bibliometric knowledge with medical practice, scientific administrators to adjust the direction of research and apply grants to promising research.

## Supplementary Material

Supplemental Material
